# Supplementary material for: Activity of Cerebellar Nuclei Neurons Correlates with ZebrinII Identity of Their Purkinje Cell Afferents
Source: Cells. 2021 Oct 7;10(10):2686. doi: 10.3390/cells10102686 (PMC8534335; doi:10.3390/cells10102686)
Supplement: Supplementary file 1 [file cells-10-02686-s001.zip › proofs_SupplementaryFigures_final.pdf]

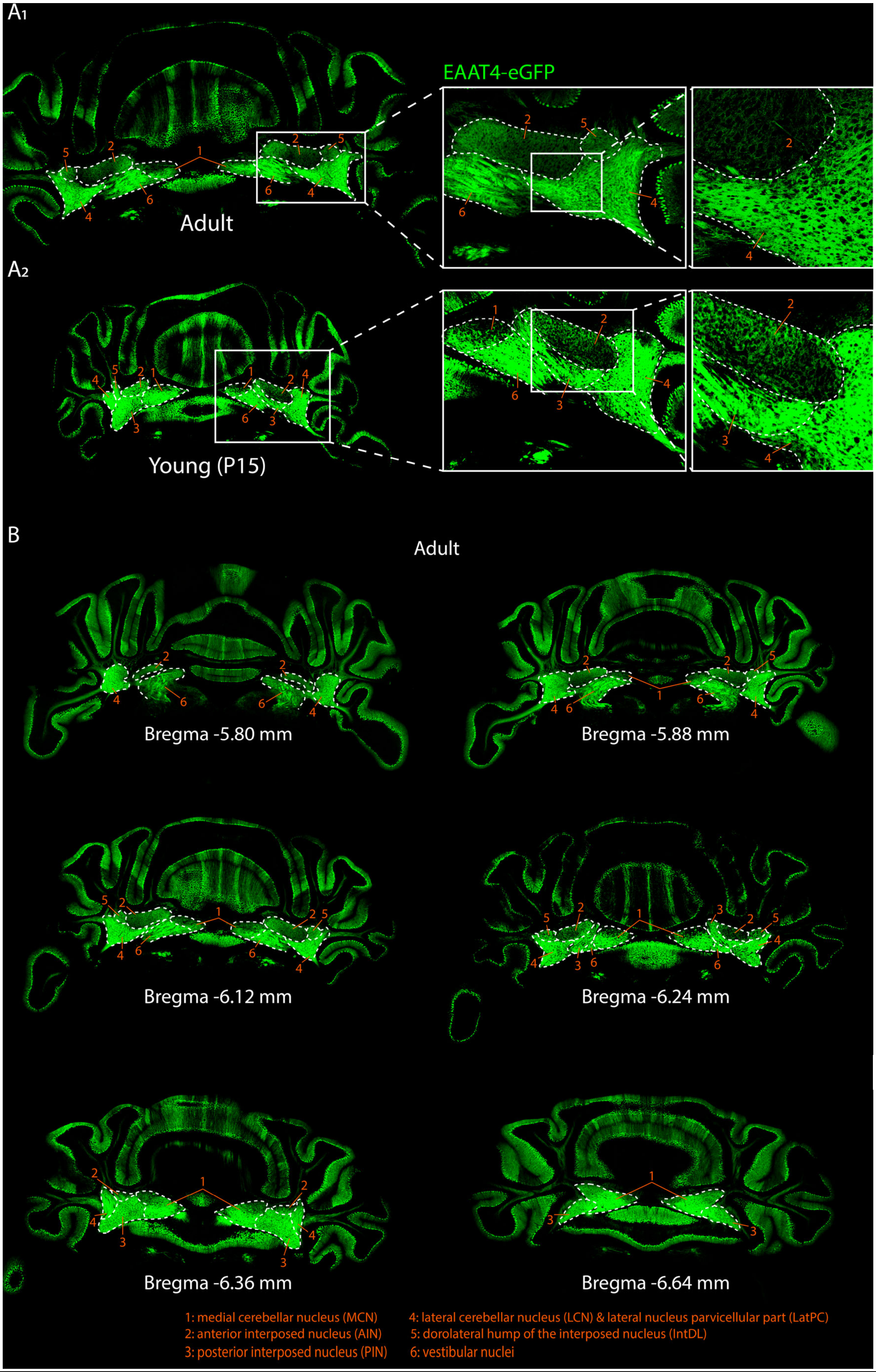
A1
EAAT4-eGFP
Adult
A2
Young (P15)
B
Adult
Bregma -5.80 mm
Bregma -5.88 mm
Bregma -6.12 mm
Bregma -6.24 mm
Bregma -6.36 mm
Bregma -6.64 mm
1: medial cerebellar nucleus (MCN)
2: anterior interposed nucleus (AIN)
3: posterior interposed nucleus (PIN)
4: lateral cerebellar nucleus (LCN) & lateral nucleus parvicellular part (LatPC)
5: dorolateral hump of the interposed nucleus (IntDL)
6: vestibular nuclei

Figure S1. Endogenous GFP expression reveals differential fluorescence in cerebellar cortical and nuclei. (**A**) Confocal tile scan of coronal sections from adult (**A**1) and P15 (**A**2) *Slc1a6-EGFP* mice. Inserts of a tile scan image to show the difference between a high (lateral nucleus; indicated by '4') versus low (anterior interposed nucleus; indicated by '2') expression pattern of the subnuclei in the CN. (**B**) Six tile scan images in which the individual nuclei are demarcated numerically (as indicated by the definitions below the figure), guided by the Paxinos and Franklin anatomical atlas [28] and the previously published ZebrinII pattern in the CN [20].

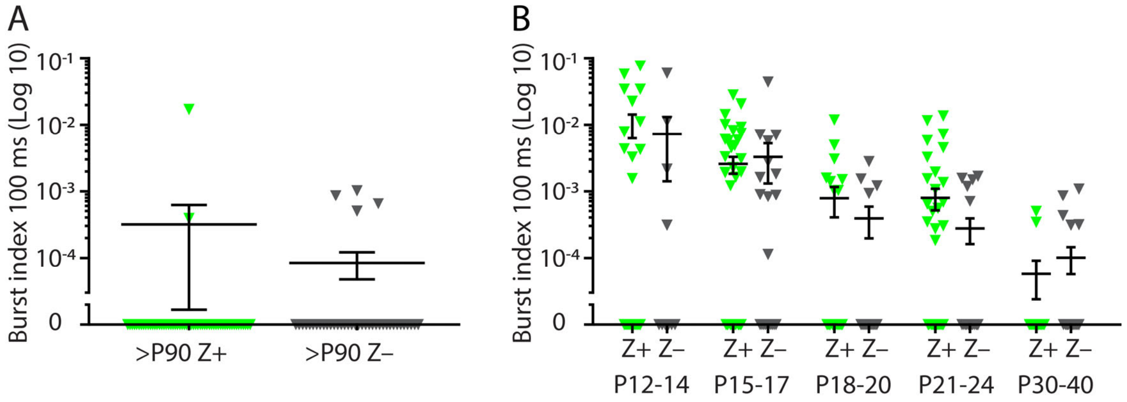

Figure S2. CN burst firing in Z+ and Z– neurons *in vivo*. (**A**) Quantification of 'burst index 100 ms', i.e., the fraction of spikes fired in bursts as defined by ≥3 spikes at ≥100 Hz followed by a pause of ≥100 ms. (**B**) Burst index 100 ms for postnatal stages. See Supplementary Table 1 for all statistical data. Data are represented as mean ± SEM.
