# Supplementary material for: Activity of Cerebellar Nuclei Neurons Correlates with ZebrinII Identity of Their Purkinje Cell Afferents
Source: Cells. 2021 Oct 7;10(10):2686. doi: 10.3390/cells10102686 (PMC8534335; doi:10.3390/cells10102686)
Supplement: Supplementary file 1 [file cells-10-02686-s001.zip › proofs_SupplementaryTables_final.pdf]

cells MDPI

Table S1. Data for *in vivo* electrophysiology in the adult mouse. Descriptive values are represented as ± SEM. Abbreviations: Z+ = ZebrinII positive domain, Z− = ZebrinII negative domain, DF = degrees of freedom, Hz = hertz.

Related Figure	Test Applied	Group	*p*-Value	Population-Value	Descriptive Values	Population Size
Figure 1C	Unpaired *t*-test	Z+ vs. Z−	**<u>0.0107</u>**	(*t*) 2.603 (DF) 95	53.9 ± 3.5 Hz vs. 68.7 ± 4.7 Hz	57 vs. 40 cells (10 mice)
Figure 1D_1	Mann–Whitney U test	Z+ vs. Z−	0.6211	(*U*) 1072	1.25 ± 0.27 vs. 1.02 ± 0.29	57 vs. 40 cells (10 mice)
Figure 1D_2	Mann–Whitney U test	Z+ vs. Z−	0.9957	(*U*) 1139	0.48 ± 0.02 vs. 0.48 ± 0.03	57 vs. 40 cells (10 mice)
Figure 1E	Mann–Whitney U test	Z+ vs. Z−	0.7041	(*U*) 1095	0.0027 ± 0.0018 vs. 0.0008 ± 0.0003	57 vs. 40 cells (10 mice)
Figure 1F	Mann–Whitney U test	Z+ vs. Z−	0.9201	(*U*) 1126	11.3 ± 3.7 vs. 7.5 ± 2.7	57 vs. 40 cells (10 mice)
Figure 1G	Mann–Whitney U test	Z+ vs. Z−	0.8568	(*U*) 1115	984.2 ± 628.6 vs. 379.6 ± 288.7	57 vs. 40 cells (10 mice)
Supplementary Figure 2A	Mann–Whitney U test	Z+ vs. Z−	0.1954	(*U*) 1066	0.0003 ± 0.0003 vs. 0.0001 ± 0.0001	57 vs. 40 cells (10 mice)
Supplementary Figure 2B	Kruskal–Wallis test	P12-P14: Z+ vs. Z−	0.6097	(Z) 0.511	0.0103 ± 0.0040 vs. 0.0073 ± 0.0059	25 vs. 10 cells (7 mice)
		P15-P17: Z+ vs. Z−	0.3305	(Z) 0.973	0.0026 ± 0.0007 vs. 0.0033 ± 0.0020	55 vs. 22 cells (13 mice)
		P18-P20: Z+ vs. Z−	0.9240	(Z) 0.096	0.0008 ± 0.0004 vs. 0.0004 ± 0.0002	34 vs. 17 cells (9 mice)
		P21-P24: Z+ vs. Z−	0.5962	(Z) 0.530	0.0008 ± 0.0003 vs. 0.0003 ± 0.0001	70 vs. 25 cells (17 mice)
		P30-P40: Z+ vs. Z−	0.8104	(Z) 0.240	0.0001 ± 0.0001 vs. 0.0001 ± 0.0001	17 vs. 32 cells (6 mice)

Table S2. Data for *in vivo* electrophysiology in mice from postnatal day 12 to 40. Descriptive values are represented as mean ± SEM. Abbreviations: Z+ = ZebrinII positive domain, Z– = ZebrinII negative domain, DF = degrees of freedom, Hz = hertz.

Related Figure	Test Applied	Group	*p*-Value	Population-Value	Descriptive Values	Population Size
Figure 2B	Kruskal–Wallis test	P12-P14: Z+ vs. Z–	0.1287	(Z) 1.519	7.90 ± 1.66 Hz vs. 19.37 ± 5.15 Hz	25 vs. 10 cells (7 mice)
		P15-P17: Z+ vs. Z–	0.2028	(Z) 1.274	17.57 ± 2.42 Hz vs. 23.38 ± 3.41 Hz	55 vs. 22 cells (13 mice)
		P18-P20: Z+ vs. Z–	0.1368	(Z) 1.488	27.61 ± 3.80 Hz vs. 41.08 ± 6.70 Hz	34 vs. 17 cells (9 mice)
		P21-P24: Z+ vs. Z–	0.5107	(Z) 0.658	52.78 ± 3.25 Hz vs. 52.97 ± 8.03 Hz	70 vs. 25 cells (17 mice)
		P30-P40: Z+ vs. Z–	0.5542	(Z) 0.592	67.11 ± 5.74 Hz vs. 61.61 ± 4.92 Hz	17 vs. 32 cells (6 mice)
Figure $2C_1$	Kruskal–Wallis test	P12-P14: Z+ vs. Z–	0.6442	(Z) 0.462	1.83 ± 0.21 vs. 1.47 ± 0.21	25 vs. 10 cells (7 mice)
		P15-P17: Z+ vs. Z–	0.0082	(Z) 2.645	1.77 ± 0.21 vs. 1.00 ± 0.13	55 vs. 22 cells (13 mice)
		P18-P20: Z+ vs. Z–	0.7312	(Z) 0.344	1.54 ± 0.40 vs. 2.08 ± 0.66	34 vs. 17 cells (9 mice)
		P21-P24: Z+ vs. Z–	0.6427	(Z) 0.464	1.19 ± 0.21 vs. 0.99 ± 0.19	70 vs. 25 cells (17 mice)
		P30-P40: Z+ vs. Z–	0.9670	(Z) 0.041	0.65 ± 0.06 vs. 0.74 ± 0.12	17 vs. 32 cells (6 mice)
Figure $2C_2$	Brown-Forsythe and Welch ANOVA test	P12-P14: Z+ vs. Z–	0.4785	(*t*) 0.724 (DF) 17.92	0.87 ± 0.06 vs. 0.79 ± 0.09	25 vs. 10 cells (7 mice)
		P15-P17: Z+ vs. Z–	0.0267	(*t*) 2.302 (DF) 39.50	0.69 ± 0.03 vs. 0.55 ± 0.05	55 vs. 22 cells (13 mice)
		P18-P20: Z+ vs. Z–	0.5302	(*t*) 0.634 (DF) 33.06	0.55 ± 0.04 vs. 0.50 ± 0.05	34 vs. 17 cells (9 mice)
		P21-P24: Z+ vs. Z–	0.2503	(*t*) 1.162 (DF) 52.40	0.48 ± 0.02 vs. 0.44 ± 0.03	70 vs. 25 cells (17 mice)
		P30-P40: Z+ vs. Z–	0.8360	(*t*) 0.208 (DF) 40.50	0.49 ± 0.04 vs. 0.48 ± 0.04	17 vs. 32 cells (6 mice)
Figure 2D	Kruskal–Wallis test	P12-P14: Z+ vs. Z–	0.2278	(Z) 1.206	0.0141 ± 0.0043 vs. 0.0073 ± 0.0059	25 vs. 10 cells (7 mice)
		P15-P17: Z+ vs. Z–	0.8160	(Z) 0.233	0.0043 ± 0.0001 vs. 0.0033 ± 0.0020	55 vs. 22 cells (13 mice)
		P18-P20: Z+ vs. Z–	0.2269	(Z) 1.208	0.0017 ± 0.0006 vs. 0.0004 ± 0.0002	34 vs. 17 cells (9 mice)
		P21-P24: Z+ vs. Z–	**0.0052**	(Z) 2.792	0.0026 ± 0.0006 vs. 0.0003 ± 0.0001	70 vs. 25 cells (17 mice)
		P30-P40: Z+ vs. Z–	**0.0054**	(Z) 2.782	0.0032 ± 0.0013 vs. 0.0001 ± 0.0001	17 vs. 32 cells (6 mice)
Firing frequency	Unpaired *t*-test	Z–: P90 vs. P30-40	0.3019	(*t*) 1.040 (DF) 70	68.70 ± 4.66 Hz vs. 61.61 ± 4.92 Hz	40 vs. 32 cells (16 mice)
		Z+: P90 vs. P30-40	0.0663	(*t*) 1.865 (DF) 72	53.89 ± 3.47 Hz vs. 67.11 ± 5.74 Hz	57 vs. 17 cells (16 mice)
CV	Mann–Whitney U test	Z–: P90 vs. P30-40	0.4953	(U) 579	1.023 ± 0.29 vs. 0.74 ± 0.12	40 vs. 32 cells (16 mice)
		Z+: P90 vs. P30-40	0.6267	(U) 446	1.25 ± vs. 0.65 ± 0.06	57 vs. 17 cells (16 mice)
CV2	Unpaired *t*-test	Z–: P90 vs. P30-40	0.8456	(*t*) 0.195 (DF) 70	0.48 ± 0.03 vs. 0.48 ± 0.04	40 vs. 32 cells (16 mice)
	Mann–Whitney U test	Z+: P90 vs. P30-40	0.7972	(U) 464	0.48 ± 0.02 vs. 0.49 ± 0.04	57 vs. 17 cells (16 mice)
Burst index 50ms	Mann–Whitney U test	Z–: P90 vs. P30-40	**0.0145**	(U) 3	0.0008 ± 0.0003 vs. 0.0001 ± 0.0001	40 vs. 32 cells (16 mice)
		Z+: P90 vs. P30-40	0.0790	(U) 365	0.0027 ± 0.0017 vs. 0.0032 ± 0.0013	57 vs. 17 cells (16 mice)

Table S3. Data for *in vitro* electrophysiology in adult mice. Descriptive values are represented as mean ± SEM. Abbreviations: V_m = membrane potential, pA = picoampere, Z+ = ZebrinII positive, Z– = ZebrinII negative, DF = degrees of freedom, Hz = hertz, ISI = interspike interval.

Related Figure	Test Applied	Group	*p*-Value	Population-Value	Descriptive Values	Population Size
Figure 3B	Unpaired *t*-test	Z+ vs. Z–	0.4633	(*t*) 0.742 (DF) 32	−48.2 ± 1.0 V_m vs. −47.2 ± 0.7 V_m	20 vs. 14 cells (22 mice)
Figure 3C	Mann–Whitney U test	Z+ vs. Z–	0.4363	(*U*) 117	70.8 ± 9.1 Hz vs. 83.9 ± 10.0 Hz	20 vs. 14 cells (22 mice)
Figure $3D_1$	Mann–Whitney U test	Z+ vs. Z–	0.7692	(*U*) 131	0.17 ± 0.04 vs. 0.13 ± 0.02	20 vs. 14 cells (22 mice)
Figure $3D_2$	Mann–Whitney U test	Z+ vs. Z–	0.8698	(*U*) 135	0.05 ± 0.01 vs. 0.5 ± 0.01	20 vs. 14 cells (22 mice)
Figure 3F	Two-way ANOVA	Effect of ZebrinII	0.2687	F (1, 16) = 1.313		9 vs. 9 cells (15 mice)
		Effect of current input	**<u><0.0001</u>**	F (11, 176) = 398.2		
		Effect of ZebrinII * current input	0.6119	F (11, 176) = 0.828		
		−100 pA: Z+ vs. Z–	>0.9999	(*t*) 0.000 (DF) 192	0.00 Hz vs. 0.00 Hz	
		−50 pA: Z+ vs. Z–	>0.9999	(*t*) 0.000 (DF) 192	0.00 Hz vs. 0.00 Hz	
		0 pA: Z+ vs. Z–	0.6980	(*t*) 0.389 (DF)192	0.53 ± 0.48 Hz vs. 6.25 ± 5.51 Hz	
		50 pA: Z+ vs. Z–	0.3735	(*t*) 0.892 (DF) 192	34.7 ± 8.2 Hz vs. 47.9 ± 5.0 Hz	
		100 pA: Z+ vs. Z–	0.5248	(*t*) 0.637 (DF) 192	69.9 ± 11.6 Hz vs. 79.3 ± 7.3 Hz	
		150 pA: Z+ vs. Z–	0.2470	(*t*) 1.161 (DF) 192	93.3 ± 10.2 Hz vs. 110.4 ± 9.4 Hz	
		200 pA: Z+ vs. Z–	0.2811	(*t*) 1.081 (DF) 192	122.2 ± 12.9 Hz vs. 138.1 ± 0.6 Hz	
		250 pA: Z+ vs. Z–	0.2108	(*t*) 1.256 (DF) 192	144.2 ± 12.5 Hz vs. 162.6 ± 11.4 Hz	
		300 pA: Z+ vs. Z–	0.1948	(*t*) 1.301 (DF) 192	169.5 ± 14.3 Hz vs. 188.6 ± 12.2 Hz	
		350 pA: Z+ vs. Z–	0.1707	(*t*) 1.375 (DF) 192	192.0 ± 15.4 Hz vs. 212.2 ± 12.4 Hz	
		400 pA: Z+ vs. Z–	0.1338	(*t*) 1.506 (DF) 192	213.1 ± 16.0 Hz vs. 235.3 ± 11.6 Hz	
		450 pA: Z+ vs. Z–	0.1166	(*t*) 1.576 (DF) 192	231.7 ± 15.9 Hz vs. 254.9 ± 12.0 Hz	
Figure 3G	Mixed-effects analysis	Effect of ZebrinII	**<u>0.7614</u>**	F (19,285) = 5.912		8 vs. 9 cells (14 mice)
		Effect of ISI	**<u><0.0001</u>**	F (1, 15) = 0.956		
		Effect of ZebrinII * ISI	>0.9999	F (19, 285) = 0.199		
		ISI 1	>0.9999	(*t*) 0.000 (DF) 300	1.000 vs. 1.000	
		ISI 2	0.6722	(*t*) 0.424 (DF) 300	0.794 ± 0.024 vs. 0.850 ± 0.065	
		ISI 3	0.5772	(*t*) 0.558 (DF) 300	0.766 ± 0.039 vs. 0.839 ± 0.084	
		ISI 4	0.8290	(*t*) 0.216 (DF) 300	0.765 ± 0.041 vs. 0.793 ± 0.099	
		ISI 5	0.8472	(*t*) 0.193 (DF) 300	0.752 ± 0.052 vs. 0.777 ± 0.089	
		ISI 6	0.6575	(*t*) 0.444 (DF) 300	0.740 ± 0.048 vs. 0.798 ± 0.099	
		ISI 7	0.6786	(*t*) 0.415 (DF) 300	0.744 ± 0.054 vs. 0.798 ± 0.123	
		ISI 8	0.6331	(*t*) 0.478 (DF) 300	0.741 ± 0.053 vs. 0.804 ± 0.118	
		ISI 9	0.8170	(*t*) 0.232 (DF) 300	0.734 ± 0.057 vs. 0.765 ± 0.089	
		ISI 10	0.7172	(*t*) 0.363 (DF) 300	0.725 ± 0.063 vs. 0.772 ± 0.112	
		ISI 11	0.7325	(*t*) 0.342 (DF) 300	0.733 ± 0.053 vs. 0.778 ± 0.120	
		ISI 12	0.8080	(*t*) 0.243 (DF) 300	0.754 ± 0.056 vs. 0.786 ± 0.114	
		ISI 13	0.7920	(*t*) 0.264 (DF) 300	0.769 ± 0.070 vs. 0.804 ± 0.112	
		ISI 14	0.9006	(*t*) 0.125 (DF) 300	0.778 ± 0.063 vs. 0.795 ± 0.124	
		ISI 15	0.7658	(*t*) 0.298 (DF) 300	0.745 ± 0.057 vs. 0.784 ± 0.115	
		ISI 16	0.8018	(*t*) 0.251 (DF) 300	0.769 ± 0.070 vs. 0.802 ± 0.136	
		ISI 17	0.6176	(*t*) 0.500 (DF) 300	0.750 ± 0.059 vs. 0.815 ± 0.147	
		ISI 18	0.8231	(*t*) 0.224 (DF) 300	0.781 ± 0.067 vs. 0.810 ± 0.151	
		ISI 19	0.9560	(*t*) 0.055 (DF) 300	0.790 ± 0.068 vs. 0.797 ± 0.121	
		ISI 20	0.8189	(*t*) 0.229 (DF) 300	0.780 ± 0.067 vs. 0.810 ± 0.145	

Table S4. Data for action potential properties in adult mice, acquired with *in vitro* electrophysiology. Descriptive values are represented as mean ± SEM. Abbreviations: V_m = membrane potential, mV = millivolt, ms = millisecond, pA = picoampere, Z+ = ZebrinII positive domain, Z– = ZebrinII negative domain, DF = degrees of freedom, Hz = hertz.

Related figure	Test applied	Group	*p*-value	Population-Value	Descriptive values	Population size
Action potential threshold	Unpaired *t*–test	Z+ vs. Z–	0.6628	(t) 2.603 (DF) 15	−40.09 ± 2.65 mV vs. −41.58 ± 2.11 mV	8 vs. 9 cells (13 mice)
Peak maximum	Unpaired *t*–test	Z+ vs. Z–	0.7640	(t) 0.306 (DF) 15	10.48 ± 5.281 mV vs. 8.12 ± 5.55 mV	8 vs. 9 cells (13 mice)
Peak amplitude	Unpaired *t*–test	Z+ vs. Z–	0.8666	(t) 0.171 (DF) 15	62.02 ± 4.42 mV vs. 60.75 ± 5.77 mV	8 vs. 9 cells (13 mice)
Half-width	Unpaired *t*–test	Z+ vs. Z–	0.5258	(t) 0.650 (DF) 15	0.29 ± 0.02 ms vs. 0.27 ± 0.02 ms	8 vs. 9 cells (13 mice)
Rise time	Unpaired *t*–test	Z+ vs. Z–	0.9938	(t) 0.008 (DF) 15	0.24 ± 0.02 ms vs. 0.24 ± 0.03 ms	8 vs. 9 cells (13 mice)
Decay time	Unpaired *t*–test	Z+ vs. Z–	0.9914	(t) 0.011 (DF) 15	0.22 ± 0.02 ms vs. 0.24 ± 0.02 ms	8 vs. 9 cells (13 mice)
Fast after-hyperpolarization	Unpaired *t*–test	Z+ vs. Z–	0.2936	(t) 2.057 (DF) 15	10.31 ± 1.07 mV vs. 8.06 ± 1.70 mV	8 vs. 9 cells (13 mice)
Rheobase	Unpaired *t*-test	Z+ vs. Z–	0.9009	(t) 0.127 (DF) 15	37.50 ± 8.18 pA vs. 38.89 ± 7.35 pA	8 vs. 9 cells (13 mice)

Table S5. Data for CN neuron morphology in adult mice. Descriptive values are represented as mean ± SEM. Abbreviations: µm = micrometer, µm^2 = square micrometer, Z+ = ZebrinII positive, Z− = ZebrinII negative, DF = degrees of freedom.

Related Figure	Test Applied	Group	*p*-Value	Population-Value	Descriptive Values	Population Size
Figure 4B$_1$	Welch's *t*-test	Z+ vs. Z−	0.3121	(*t*) 1.034 (DF) 22.	306.4 ± 16.69 µm^2 vs. 342.1 ± 30.16 µm^2	17 vs. 15 cells (19 mice)
Figure 4B$_2$	Mixed-effects analysis	Effect of distance from soma	**<0.0001**	F (13, 272) = 21.83		16 vs. 9 cells (16 mice)
		Effect of ZebrinII	0.3675	F (1, 23) = 0.845		
		Effect of distance * ZebrinII	0.7919	F (13, 272) = 0.670		
		15 µm: Z+ vs. Z−	0.8081	(*t*) 0.243 (DF) 295	4.56 ± 0.46 vs. 4.78 ± 0.15	
		25 µm: Z+ vs. Z−	0.4381	(*t*) 0.776 (DF) 295	5.31 ± 0.50 vs. 6.00 ± 0.50	
		35 µm: Z+ vs. Z−	0.6780	(*t*) 0.416 (DF) 295	5.19 ± 0.53 vs. 5.56 ± 0.67	
		45 µm: Z+ vs. Z−	0.3242	(*t*) 0.988 (DF) 295	4.73 ± 0.66 vs. 5.56 ± 0.75	
		55 µm: Z+ vs. Z−	0.4906	(*t*) 0.690 (DF) 295	4.50 ± 0.76 vs. 5.11 ± 0.99	
		65 µm: Z+ vs. Z−	0.5611	(*t*) 0.582 (DF) 295	4.06 ± 0.67 vs. 3.75 ± 0.65	
		75 µm: Z+ vs. Z−	0.6836	(*t*) 0.408 (DF) 295	4.50 ± 0.75 vs. 4.78 ± 1.32	
		85 µm: Z+ vs. Z−	0.9592	(*t*) 0.051 (DF) 295	3.62 ± 0.62 vs. 3.44 ± 0.50	
		95 µm: Z+ vs. Z−	0.6346	(*t*) 0.476 (DF) 295	2.71 ± 0.49 vs. 3.13 ± 1.08	
		105 µm: Z+ vs. Z−	0.6039	(*t*) 0.519 (DF) 295	2.25 ± 0.49 vs. 2.38 ± 1.08	
		115 µm: Z+ vs. Z−	0.2434	(*t*) 1.169 (DF) 295	1.46 ± 0.33 vs. 2.43 ± 0.72	
		125 µm: Z+ vs. Z−	0.9940	(*t*) 0.007 (DF) 295	1.67 ± 0.57 vs. 1.63 ± 0.57	
		135 µm: Z+ vs. Z−	**0.0288**	(*t*) 2.196 (DF) 295	0.60 ± 0.21 vs. 2.63 ± 0.91	
		145 µm: Z+ vs. Z−	0.3316	(*t*) 0.973 (DF) 295	0.25 ± 0.11 vs. 1.11 ± 0.42	
Soma size perimeter	Unpaired *t*-test	Z+ vs. Z−	0.6010	(*t*) 0.529 (DF) 30	70.26 ± 2.14 µm vs. 72.49 ± 3.79 µm	17 vs. 15 cells (19 mice)
